# Harnessing Indonesia’s biodiversity for sustainable water treatment: a review of local plant-based solutions

**DOI:** 10.1007/s11356-025-36485-2

**Published:** 2025-05-05

**Authors:** Muhamad Imaduddin, Ingo Eilks

**Affiliations:** 1 Department of Biology and Chemistry, Institute for Science Education (IDN), University of Bremen, Leobener Str. NW2, 28359 Bremen, Germany; 2 Institut Agama Islam Negeri Kudus, Conge Ngembalrejo St. Bae, PO BOX 51, Kudus, 59322 Central Java Indonesia; 3 Faculty of Mathematics and Science, Universitas Negeri Malang, Semarang St. No. 5, Malang, 65145 East Java Indonesia

**Keywords:** Wastewater, Wastewater treatment, Plant-based water treatment, Local plants, Indonesia

## Abstract

Access to clean water is a critical global issue, with millions of people facing significant challenges, particularly in Southeast Asia. Recent research has increasingly focused on Indonesia’s rich biodiversity to develop environmentally friendly water purification methods using local plant materials. This approach offers a promising alternative to artificial water treatment solutions. This paper reviews the literature regarding using Indonesia’s local plants for water treatment. The analysis highlights three main aspects: the local plants that might be utilized, the mechanisms involved in the treatment process, and the types of treated water. The local plants considered encompass aquatic and wetland plants, fruit plants, fiber plants, grain plants, medicinal and ornamental plants, timber and latex-producing trees, as well as vegetables and food crops. The mechanisms involved in water treatment using Indonesia’s local plants include adsorption, coagulation-flocculation, membrane filtration, and phytoremediation. The types of treated water encompass challenging raw water such as peat water, wetland saline water, river, and well water, along with various forms of wastewater, including domestic wastewater, aquaculture effluent, effluent from tofu-tempeh and tapioca factories, textile industry wastewater, dye waste from the batik industry, wastewater containing heavy metals, and effluent from oil and gas factories. Further investigation is essential, particularly to expand upon laboratory results from recent years, enabling these methods to address the issue of clean water scarcity effectively.

## Introduction

Access to safe water is a fundamental human need. A UN report has highlighted the significant challenges faced by 2 billion people globally in accessing this basic necessity. Southeast Asia is one of the regions most affected by water insecurity, with 110 million individuals facing problems with clean water supply despite the region’s progress in the water sector (Geall 2019). Even though 75% of the earth is covered by water, access to clean water is still limited, especially in developing countries like Indonesia. Indonesia, with approximately 18,000 islands, has recently experienced rapid economic growth, with an annual increase of 6% and a GDP of approximately $3000 (World Bank Group 2024). Nevertheless, issues such as sanitation and access to clean water still require attention.

Indonesia, with a population of approximately 275 million people, is the fourth-largest country in the world and has the largest economy in Southeast Asia. However, it faces significant challenges related to water access and sanitation. In contrast, Malaysia, which is one of Indonesia’s closest neighbors, enjoys 94% access to safe drinking water. Indonesia, on the other hand, has only 30% access to safe drinking water and similarly falls behind in sanitation as well (UN-Water 2024a, b). This stark contrast highlights Indonesia’s urgent need to improve its clean water infrastructure and sanitation systems to keep pace with other Southeast Asian nations with similar geographical conditions. Moreover, monitoring at 563 points across 34 provinces in Indonesia from 2015 to 2020 revealed that 65.30% of river water was classified as heavily polluted (Ministry of Environment and Forestry-Indonesia et al., 2021). This pollution not only possibly threatens public health, leading to various health issues across regions, but also contributes to economic losses in tourism, fisheries, housing, irrigation, and drinking water supply.

The issue of water scarcity is urgent due to the increasing demand for this vital resource. Consequently, the necessity for water purification and waste disposal has become a significant research topic in recent years to develop sustainable, eco-friendly, and cost-effective water purification and recycling methods. Various pollutants are found in water, including heavy metal ions, high-concentration salts, microbes, oil, petroleum byproducts, plastic waste, organic dyes, and pharmaceuticals. The accumulation of these pollutants in water significantly threatens human health and other organisms. Over the years, various methods have been proposed for removing pollutants from water, encompassing physical, chemical, and biological processes. Technologies such as filtration (Nawi et al. 2022; Abdiyev et al. 2023), adsorption (Elgarahy et al. 2021; Varghese et al. 2022), coagulation/flocculation (Mao et al. 2024; Usman et al. 2023), and phytoremediation (Wu et al. 2023; Retta et al. 2023) have been implemented.

The use of plant-based materials in the treatment of water and wastewater is receiving growing attention in the field of applied polymer science. Over the past decade, numerous studies on wastewater treatment have been published in polymer science journals, emphasizing the focus on these materials (Islam et al. 2024). Research is ongoing to replace synthetic compounds with natural ingredients or modify natural materials to minimize synthetic content. Some studies even combine two natural raw materials to enhance performance (Usman et al. 2023). This shift highlights the significant potential of plant biodiversity in overcoming water treatment challenges. The connection between biodiversity and sustainable wastewater treatment technologies is exemplified by the use of biomass as a raw material for producing sustainable bio-based materials (SBMs). Utilizing biomass aligns with the principles of sustainability by reducing costs and environmental impacts while also making use of existing natural resources (Wu et al. 2022). A literature review by Mishra et al. (2024) specifically discussed how biodiversity, in this case, is the abundance of cellulose, which becomes a valuable product for wastewater treatment. There is increasing interest in developing sustainable and efficient materials for wastewater treatment to address wastewater treatment issues. The use of these natural materials supports the principle of sustainability by reducing dependence on hazardous chemicals and increasing the efficiency of wastewater treatment (Ahsan et al. 2001).

Indonesia, known for its rich biodiversity and major agricultural production, has a significant opportunity to enhance sustainable water and wastewater treatment solutions. Approximately 29% of Indonesia’s 190 million hectares of land is used for agriculture (Quincieu 2015), making it one of the largest tropical fruit producers in the ASEAN region (Food and Agriculture Organization of the United Nations 2023). The variety of local Indonesian plants includes thousands of species, each comprising tens to hundreds of subspecies or varieties (Thomson et al. 2007). This paper reviews the potential of Indonesia’s local plants in the process and efforts of water and wastewater treatment. Of course, in this case, Indonesia’s rich biodiversity is utilized to reveal the potential for developing innovative and sustainable solutions to global challenges related to clean water.

## Method

### Data collection

#### Search strategy

A comprehensive and methodical examination of the literature was conducted, and data pertinent to water treatment was gathered from many indexing databases. It is recommended that researchers employ a multitude of search engines to achieve optimal results, rather than relying on a single search engine (Samadzadeh et al. 2013). In light of the popularity of two indexers of scientific publications on topics encompassing science, engineering, and social sciences, the Web of Science and Scopus were employed (Mongeon and Paul-Hus 2016). In consideration of its extensive reach, researchers also utilize Google Scholar as a search engine, despite the limitations of this search engine in comparison to traditional bibliographic data. These limitations include the absence of essential functions such as chunking (word formation), proximity operators, the use of brackets, and search history (Bramer et al. 2017). However, using these three databases is considered sufficient for a systematic review that will reveal the potential of Indonesia’s local plant-based water treatment. The strategy was prepared in early May 2024 and executed on May 14, 2024.

#### Keywords

This review considers perspectives on research on Indonesia’s local plants in water and wastewater treatment. The keyword “natur*” was used to identify findings on using natural materials in water treatment, including wastewater, with the understanding that it would be sorted explicitly for plant-based processing. The potential for implementing integrative processing using several types of natural materials was also considered. Indonesian specifications were also provided, given that the expected target is natural materials abundant in the Indonesian area.

It should be noted that the data search sequence varies between databases. Specifically, the option to export metadata documents in bulk is unavailable when searching in Google Scholar. To obtain Google Scholar results, the Publish or Perish software (Harzing 2023) was used. However, this software restricts downloads to a maximum of 1000 results. Consequently, we elected to limit the search to articles published between 2014 and 2023 on the Google Scholar indexer. The detailed search queries are presented in Table 1.

**Table 1:** Databases searched, including the search strings used and the number of hits

Type of data base	Search query	Number of hits
Scopus	TITLE-ABS-KEY (“water treatment” AND “natur*” AND “Indonesia”) AND (LIMIT-TO (AFFILCOUNTRY, “Indonesia”))	42
Web of Science	“water treatment” AND “natur*” AND “Indonesia” (All Fields) and Review Article (Exclude–Document Types) and Editorial Material or Retracted Publication (Exclude–Document Types)	111
Google Scholar	“water treatment” [title], natur* AND Indonesia from 2014 to 2023, no citations, no patents	747

#### Study selection

To minimize bias, we reviewed all relevant titles and abstracts, manually excluding some using the cloud-based software Rayyan if they did not match the search terms. Table 2 outlines the criteria for including or excluding articles.

**Table 2:** Inclusion and exclusion criteria for retrieving the dataset

	Inclusion criteria	Exclusion criteria
Materials	The utilization of natural materials derived from organisms belonging to the kingdom of Plantae	The absence of the use of natural materials derived from plants
Types of evidence sources	1) English-only articles 2) Peer-reviewed journal articles, book chapters, and conference papers 3) Articles that can be accessed with complete metadata links 4) Empirical research	1) Articles presented in non-English or partial English 2) Books, reports, newspapers, bulletins, magazines, reports, papers without sufficient references, and other grey literature 3) Articles with incomplete access links 4) Research exploring data taken from document content such as books, regulations, online metadata, and social media is excluded 5) Non-empirical research
Setting	The research uses natural plant materials originating from Indonesian locations	Does not indicate specifications for obtaining natural materials or research locations

### Data management and analysis

The reference details, including abstracts, were imported into Zotero 6 for Windows (Corporation for Digital Scholarship 2023) and then exported to Rayyan (Valizadeh et al. 2022) for title, abstract, and full-text screening. Documents meeting the inclusion criteria were categorized by their characteristics. For water treatment-related documents, plant material use was identified by the plant part, type of water or wastewater processed, key research findings on water conditions, and treatment mechanisms. Following this, coding was performed based on the established categories related to the type of plants, the type of treated water, and the mechanisms that may occur in the treatment process. Coding was conducted using Atlas.ti 25 and visualized with the aid of https://sankeymatic.com/.

## Finding

**Fig. 1:**
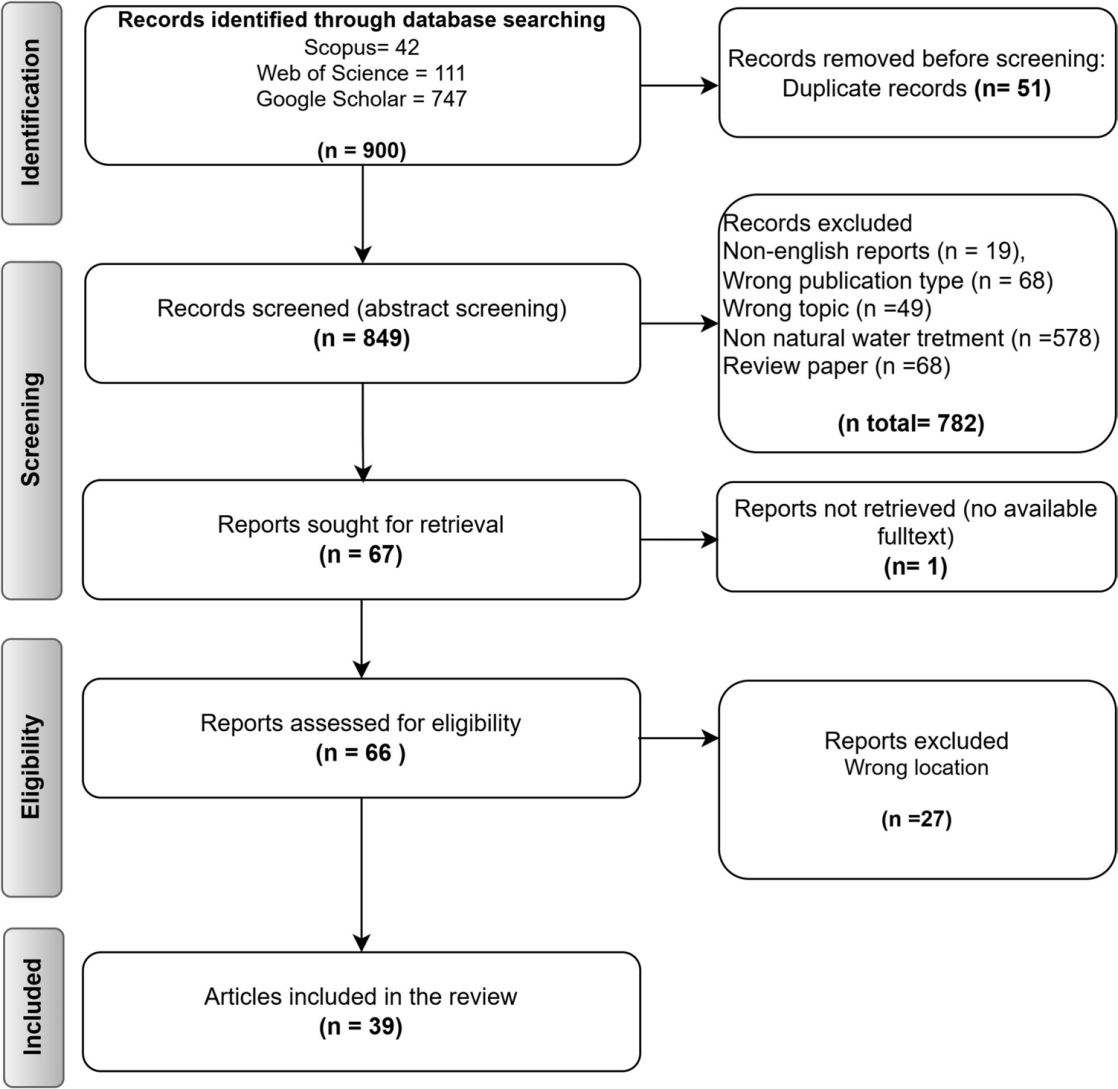
Flow diagram of the search strategy

A total of 39 academic documents that met the inclusion criteria were subjected to further analysis. These included 39 documents on water treatment (32 journal articles and 7 conference proceedings). The document acquisition process is illustrated in Fig. 1.

Numerous documents highlight the potential of Indonesia’s local plants, which are utilized through various plant parts. These plants involve different water treatment mechanisms, including adsorption, coagulation-flocculation, filtration, and phytoremediation. The variations in water treatment are demonstrated by the types of water or wastewater being improved using various water quality parameters. In this review, we classify treated water into two categories: challenging raw water and wastewater. Challenging raw water includes natural water sources with complex characteristics that make treatment difficult, such as peat water, wetland saltwater, and turbid river water. These sources are usually high in organic matter, salinity, or suspended particles, which require advanced treatment processes. Wastewater, on the other hand, originates from domestic, industrial, or agricultural activities and contains pollutants that must be removed before discharge or reuse. This distinction is important because while both require treatment, challenging raw water primarily undergoes purification for direct use, while wastewater treatment focuses on contaminant removal and environmental protection. Tables 3 and 4 provide a detailed analysis and grouping of the potential of Indonesia’s local plants in the water treatment process. These 39 documents are all marked with an asterisk (*) in the reference list.

**Table 3:** Studies on plant-based water treatment for addressing challenging raw water

Local plant (scientific name)*	Parts of plant	Source of challenging raw water	Potential uses of plant-based water treatment (PbWT)	Author (year)
**Adsorption (*N* = 1)**				
Areca palm (*Areca catechu* L.)	Areca fiber	Well water from Jambi Province (Sumatra)	Carbonized areca nut fiber, processed at 400 °C and ground to 200 mesh size, effectively enhances well water quality. It reduces odor, color, pH, TDS, TSS, and *E. coli* levels under specific conditions: 1.25 g of the biosorbent at 50 °C with a stirring speed of 150 rpm for 30 min. This biosorbent presents a cost-effective, easy, and environmentally friendly solution for wastewater treatment	Novallyan et al. (2021)
**Coagulation-flocculation (*N* = 9)**				
Aloe vera (*Aloe vera* (L.) Burm.f.)	Gel	Water collected from the Selokan Mataram River in Yogyakarta Province (Java)	Aloe vera plants function as biocoagulants in the electroflotation-biocoagulation process for river water treatment, achieving a 97% reduction in turbidity, 82.64% reduction in TDS, and adjusting pH to 6.6. The amounts used are 1.0, 1.5, and 2 g per 200 mL of water	Putra et al. (2021a)
Papaya (*Carica papaya* L.)	Seeds	Water collected from the Bedadung River in Jember, East Java Province	Papaya seeds can act as a natural biocoagulant to reduce water turbidity, with an effective dosage of 130 ppm, decreasing turbidity levels between 15 and 110 NTU. The use of single and double-media flocculation beds also significantly affects final turbidity	Wilanda et al. (2023)
		Water collected from the Tello River in Makassar, South Sulawesi Province	Papaya seeds are an effective natural coagulant for reducing TSS in water. At a pH of 3 and a concentration of 150% of the initial TSS, they can remove up to 99.13% of TSS. The process involves rapid mixing, slow mixing, and sedimentation over 60 min	Widiyanti et al. (2023)
Sweet potato (*Ipomoea batatas* (L.) Lam.)	Leaves	Water from one of the rivers in the Padalarang Cimahi area, West Java Province	Sweet potato leaf extract can act as a natural bio-flocculant in water treatment, decreasing total hardness from 348.45 to 316.00 mg/L, a reduction of about 9.31%. It effectively binds to Ca^2^⁺ and Mg^2^⁺ ions, reducing water hardness	Rohana and Asmoro (2020)
Duckweed (*Lemna minor* L.)	All parts of the plant	Water sourced from Lake Cibuntu, West Java Province	Duckweed can effectively remove turbidity, achieving up to 100% removal in water with an initial turbidity of 13.56 NTU. However, it requires careful management, as it can increase total suspended solids by 252.11% and total organic matter by 74.85%. Thus, finding the optimal dosage is essential to minimize negative impacts on water quality	Prihatinningtyas (2020)
Laban (*Vitex pinnata* L.)	Wood	Water from groundwater in Sleman Regency, Yogyakarta Province (Java)	Laban wood-activated charcoal is an effective groundwater filtration medium, reducing iron levels by 87.27% and lowering total dissolved solids (TDS) to 140 ppm. Chemical activation with H₂SO₄ for 24 h lowers iron levels to 2.27 mg/L, meeting Indonesian clean water standards	Fenditasari et al. (2019)
Mung bean (*Vigna radiata* (L.) R.Wilczek)	Seeds	Artificial peat water	Mung bean flour acts as a natural coagulant in the electroflotation-biocoagulation process for treating peat water, achieving an 84.5% reduction in turbidity and a 70.2% decrease in total dissolved solids while raising the pH from 2.7 to 6.8. The process used graphite electrodes as the anode and stainless steel as the cathode, with a direct current voltage of 20 V over 30 min	Putra et al. (2021b)
Soybean (*Glycine max* (L.) Merr.)	Seeds		Soybean extract has the potential to be a natural coagulant for reducing turbidity and color in peat water. The NaCl-extracted solution from glycine max L, at an optimal dosage of 4 mL per 500 mL of water, effectively lowers both turbidity and color	Maulidya and Putra (2020)
Moringa (*Moringa oleifera* Lam.)	Seeds	Groundwater samples from a well in Pamulang, Banten Province (Java)	Moringa seeds are effective natural coagulants, reducing groundwater turbidity by up to 97.5%	Hendrawati et al. (2016)
**Membrane filtration (*N* = 4)**				
Coconut (*Cocos nucifera* L.)	Fiber	Water collected from the Cibanten River, Banten Province (Java)	Biofiltering with coconut fiber and active zeolite shows promising results in water treatment. It reduces TDS to 249 ppm in sample 2 and 185 ppm in sample 3, while TSS drops from 1048 ppm to 9–115 ppm. The technology raises the pH to 7.23 in sample 3 and 8.5 in sample 2, and it decreases turbidity by 99.7%, BOD to 87.1%, and COD to 38.5% in sample 3	Yudanto et al. (2016)
Rice plant (*Oryza sativa* L.)	Husk	Water collected from the Gasing River in Banyuasin, South Sumatera Province	Rice husk can enhance ceramic membranes used in wastewater treatment. Membranes with 10% rice husk have achieved reductions in river water contaminants: Fe by 92.18%, Mn by 89.23%, and Zn by 99.80%	Sisnayati et al. (2019)
Banana (*Musa* spp.)	Banana peels	Peat water collected from Sukamaju Village, and saline water collected from Muara Halayung Village, both located in South Kalimantan Province	Pectin from banana peel can be used to create silica-based ultrafiltration membranes for treating swamp and brackish water. At pressures of 0.5 to 1.5 bar, the membrane flux in swamp water increased from 5.07 to 18.90 kg/m^2^/h, with organic matter rejection rates of 84.68 to 85.56%. In brackish water, the flux rose from 7.04 to 20.52 kg/m^2^/h, with rejection rates between 78.11 and 82.50%	Elma et al. (2022)
Oil palm (*Elaeis guineensis* Jacq.)	Oil palm empty fruit bunch	Artificial peat water	Cellulose fibers from oil palm empty fruit bunches can improve filtration membranes for water treatment. Polyvinylidene fluoride membranes with 1% microcrystalline cellulose showed over 90% humic acid rejection and a better flux recovery ratio than other membranes	Pramono et al. (2022)
**Phytoremediation (*N* = 1)**				
Watermilfoil (*Myriophyllum verticillatum* L.) Vetiver (*Chrysopogon zizanioides* (L.) Roberty) Heliconia (*Heliconia densiflora* Verlot)	Living plant	Water collected from the Cibuntu Lake, West Java Province	Heliconia and Vetiver planted in floating treatment wetlands (FTWs) are more effective in reducing nutrients and suspended solids than Watermilfoil. Both FTWs can improve water quality and support the long-term stability of urban lakes	Henny et al. (2019)

*Scientific names are verified through database resources on websites (1) https://powo.science.kew.org/, (2) https://identify.plantnet.org/, and (3) https://www.gbif.org/. *TDS* total dissolved solids, *TSS* total suspended solids, *BOD* biochemical oxygen demand, *COD* chemical oxygen demand, *NTU* nephelometric turbidity units, *FNU* formazin nephelometric unit

**Table 4:** Studies on plant-based water treatment for addressing wastewater

Local plant (scientific name)*	Parts of plant	Source of wastewater	Potential uses of plant-based water treatment (PbWT)	Author (year)
**Adsorption (*N* = 8)**				
Water hyacinth (*Eichhornia crassipes* (Mart.) Solms)	Stems and leaves	The wastewater generated from the production of *tempeh*, a traditional Indonesian food made from fermented soybeans, at a factory in Palembang, South Sumatra Province	Water hyacinth can be transformed into activated carbon to treat tempeh wastewater. With 4.5 g of adsorbent, turbidity can be reduced from 520.5 to 2.47 NTU (99.5% reduction) and pH increased from 3.7 to 6.4 in 150 min. Langmuir analysis shows a maximum adsorption capacity of 8.26 to 10.61 mg/g	Cundari et al. (2023)
Agarwood (*Aquilaria malaccensis* Lam.)	Wood	Liquid waste from the production of *Jumputan* fabric (a traditional textile involving the tying of parts of the fabric to resist dye, resulting in unique patterns similar to the tie-dye method) in Palembang, South Sumatra Province	Coal bottom ash and agarwood effectively treat *Jumputan* liquid waste, reducing BOD to 5.98 mg/L, COD to 15 mg/L, TSS to 22.3 mg/L, and pH to 7.32. This method also lowers color to 5 Pt–Co at a flow rate of 1 L/min over 120 min, meeting clean water quality standards and minimizing dye-related visual pollution	Hartati et al. (2021)
Chinese Cabbage (*Brassica rapa* subsp. *pekinensis* (Lour.) Hanelt)	Stems	Artificial wastewater containing phenol	Chinese cabbage stems can serve as biosorbents for removing phenol from wastewater. The optimal adsorption capacity is 0.097 mg/g at a pH of 8, with a contact time of 20 min, a biosorbent mass of 0.8 g, and an initial phenol concentration of 10 mg/L. This method presents an environmentally friendly and cost-effective solution for water treatment, particularly in regions with abundant agricultural waste	Alni et al. (2019)
Rubber (*Hevea brasiliensis* (Willd. ex A.Juss.) Müll.Arg.)	Seeds	Artificial wastewater containing methylene blue	Modified natural rubber seeds showed a maximum adsorption capacity of 784.31 mg/g for methylene blue removal in aqueous solutions at a pH of 7 to 12. This was achieved with an 800 mg/L concentration over 120 min. The process was endothermic and spontaneous at 45 °C and 65 °C, highlighting its potential for treating dye-contaminated industrial wastewater	Zulfikar et al. (2020)
Orange (*Citrus sinensis* (L.) Osbeck)	Peel		Orange peel can be an inexpensive adsorbent for removing methylene blue dye from industrial wastewater. Optimal adsorption occurs with an adsorbent dose of 0.1 to 0.8 g, a dye concentration of 10 to 80 mg/L, and a contact time of 10 to 80 min. The process showed significant color removal within 50 min and was optimized using the Langmuir isotherm model	Zainol et al. (2022)
Banana (*Musa* spp.)	Fruit bunch	Artificial wastewater containing Cu(II)	Banana bunches can be converted into activated carbon with a surface area of 33.43 m^2^/g using a 20% NaOH solution. This carbon effectively absorbs Cu(II) ions from water, following the Freundlich isotherm and pseudo-second-order kinetics (*R*^2^ > 0.9). It is a cost-effective and eco-friendly adsorbent with potential for wastewater treatment of heavy metals	Allwar et al. (2019)
Kusambi (*Schleichera oleosa* (Lour.) Oken)	Wood	Artificial wastewater containing Cr(IV)	Kusambi wood is used to create graphene oxide-magnetic (GO-Fe_3_O_4_), which effectively adsorbs Cr(VI) ions. The maximum adsorption capacity is 3.197 mg/g at pH 2, 80 min contact time, and 298 K. This process follows pseudo-second-order kinetics and Langmuir isotherm models, mainly through physisorption	Neolaka et al. (2020)
Sago palm (*Metroxylon sagu* Rottb)	Bark		Sago bark showed potential as a biosorbent for removing Cr(VI), with an optimal capacity of 61.73 mg/g at pH 3, stirring at 100 rpm, a contact time of 60 min, particle size ≤ 32 µm, and an initial concentration of 1000 mg/L. The adsorption is exothermic, and regeneration efficiency is 78.35% using 0.01 M HNO_3_	Fauzia et al. (2019)
**Coagulation-floculation (*N* = 5)**				
Sweet potato (*Ipomoea batatas* (L.) Lam.)	Leaves	Artificial turbid wastewater	Sweet potato leaf extract is a promising natural coagulant and flocculant for wastewater treatment, reducing turbidity by up to 96%. An Artificial Neural Network model optimized the process, identifying optimal parameters: 10 g/L coagulant dosage, 2 min of rapid mixing, and a mixing speed of 150 rpm	Kusuma et al. (2021)
Duckweed (*Lemna minor* L.)	All parts of the plant	Artificial turbid wastewater	Duckweed is a promising natural coagulant that can reduce water turbidity by up to 92.48% with a 30 ppm dose at pH 11, effectively treating low to high turbidity levels	Prihatinningtyas (2019)
Winged bean (*Psophocarpus tetragonolobus* (L.) DC.)	Seeds	Wastewater generated from the household-scale tofu industry in Surakarta and the small-scale tapioca industry in Wonogiri, Central Java Province	Winged bean seed powder effectively processed waste from tapioca and tofu, significantly reducing turbidity. In tapioca waste, turbidity dropped from 798 to 57 FNU, and for tofu waste, it decreased from 680 to 37 FNU. Dissolved oxygen (DO) levels increased in tapioca waste from 1.20 to 5.92 ppm and in tofu waste from 1.67 to 6.61 ppm. pH levels improved from 5.8 to 6.7 for tapioca and from 4.7 to 6.7 for tofu, meeting Indonesia's waste quality standards	Istiqomah et al. (2023)
Moringa (*Moringa oleifera* Lam.)	Seeds	Wastewater samples collected from the textile industry in Karawang, West Java Province	Moringa seeds are effective natural coagulants, reducing wastewater turbidity by up to 98.6%. They also decrease wastewater conductivity and biochemical oxygen demand by 10.8% and 11.7%, respectively, while removing metal contaminants (Cd, Cr, and Mn) and coliform bacteria	Hendrawati et al. (2016)
Leucaena (*Leucaena leucocephala* (Lam.) de Wit)	Seeds	Artificial wastewater containing Congo red dye	Leucaena seeds can effectively remove up to 99.9% of color at pH 3 with a dosage of 10 mL/L, producing half the sludge volume of alum. This makes it a more eco-friendly option for water and wastewater treatment	Kristianto et al. (2019)
**Membrane filtration (*N* = 1)**				
Kapok (*Ceiba pentandra* (L.) Gaertn.)	Raw Kapok Fiber (RKF)	Water samples provided by an oil and gas company, South Sumatra Province	Kapok fiber has shown significant potential for reducing contaminants in oil industry wastewater. Raw Kapok fiber reduced TDS by 51.81%, phenol by 62.63%, and barium by 54.20%. Modified Kapok fiber with ultrafiltration achieved even greater reductions: TDS decreased by 94.31%, phenol by 84.20%, and barium by 56.23%	Rusdi et al. (2023)
**Phytoremediation (*N* = 9)**				
Parrot’s beak heliconia (*Heliconia psittacorum* L.f.)	Living plant	Domestic black and gray water collected from the area of Duta Wacana Christian University, Yogyakarta Province (Java)	Aquatic plants in vertical-constructed wetlands can effectively treat domestic wastewater, reducing BOD by 71.64%, phosphates by 50.92%, and total coliforms by 99.67%	Sutanto and Bawole (2021)
Cattail (*Typha latifolia* L.) Azolla (*Azolla pinnata* R.Br.) Duckweed (*Lemna* spp.) Papyrus) (*Cyperus papyrus L)*	Living plant	Domestic grey water from the area of Kejawan Gebang, Sukolilo, East Java Province	Plants in a vertical sub-surface flow constructed wetland can TSS by 81%, BOD by 84%, and COD by 67%, offering a simple and cost-effective solution for treating domestic wastewater	Radityaningrum and Kusuma (2017)
Bulrush (*Scirpus grossus* L.f.)	Living plant	Artificial grey water	Bulrush in the biofiltration system achieved impressive reductions in gray wastewater pollutants: 88.2% for TSS, 88% for COD, and 77.9% for Total Nitrogen, showcasing their effectiveness in enhancing water quality	Titah et al. (2016)
Mangrove (*Rhizophora mucronata* Lam.)	Living plant	Shrimp ponds wastewater (aquaculture effluent)	Mangrove in shrimp ponds can effectively reduce nitrate, phosphate, total organic matter, and total bacteria in pond wastewater. In this study, the size of the mangrove pond did not significantly affect the decrease in these parameters. Still, this plant can potentially reduce the negative impact of waste on environmental quality	Ahmad et al. (2017)
Tiger lily (*Lilium lancifolium* Thunb.)	Living plant	Raw wastewater from the boiling process of batik (a traditional Indonesian textile method) production in Jetis, East Java Province	Plants in constructed wetlands and biofilter systems demonstrate effectiveness in treating batik industry wastewater, achieving COD reductions of 72.67–86.67%, TSS reductions of 95.85–98.18%, and oil and fat reductions of 79.47–90.04%. While these systems are efficient and cost-effective, the treated water still does not meet discharge quality standards	Rahmadyanti et al. (2020)
Water hyacinth (*Eichhornia crassipes* (Mart.) Solms)	Living plant	Wastewater from the Sasirangan industry in Banjarbaru, South Kalimantan Province; Sasirangan is a traditional fabric featuring various motifs and patterns inspired by the cultural values of the Banjar people	Water hyacinth showed great potential for treating Sasirangan wastewater. With 1 kg of plants over 9 days, it reduced TSS by 71.12%. The highest reduction in COD was 88.64% using 0.5 kg of plants in 6 days. Additionally, it stabilized the wastewater’s pH to 7.32 after 15 days	Nooryaneti et al. (2023)
		Water samples from the diamond-mine tailings pond in Pumpung village, Sungai Tiung district, South Kalimantan Province	Water hyacinth was used in phytoremediation to reduce BOD from 8.9 to 3.2 mg/L and COD from 22 to 6.5 mg/L in mining wastewater. It also lowered Fe from 0.6 mg/L, Mn from 0.16 mg/L, and ammonia from 0.63 mg/L. While the process outperformed filtration, some parameters still exceeded water quality standards	Noor et al. (2020)
Vetiver (*Chrysopogon zizanioides* (L.) Roberty)	Living plant	Crude oil spilled water from the Oil and Gas Institute (Lemigas), Jakarta Province (Java)	Vetiver can reduce crude oil wastewater’s oil content by 91.39%, decrease COD by 84.60% and BOD by 84.25% after 4 weeks. These plants thrive in oil-contaminated water, increasing wet biomass from 4.8362 to 5.1070 g	Effendi et al. (2017)
Papyrus (*Cyperus papyrus* L.)	Living plant	Diesel oil	Papyrus exhibited strong potential for wastewater treatment, especially in degrading diesel oil. In a wetland system with Cyperus papyrus and bacterial inoculum, notable changes included a 40.6% increase in viscosity, a 32.7% decrease in surface tension, the formation of degradation compounds measured at 3614.7 points, and a 227.8% increase in TDS	Harbowo and Choesin (2014)
**Integrated systems and mechanisms (*N* = 2)**				
Phytoremediation: Water hyacinth (*E. crassipe*)* Adsorption: Sugarcane (*Saccharum officinarum* L.)** Rice plant (*O. sativa*)*** Bamboo (*Bambusa* spp.)****	*Living Plant **Bagasse ***Husks ****Stick	Wastewater from the batik industry in Surakarta, Sragen, and Pekalongan, Central Java Province	Water hyacinth effectively reduces TSS, color, and ammonia in batik wastewater. Significant reductions were noted in batch processes. A bamboo charcoal column reactor showed lower reductions in BOD and COD, while a rice husk reactor achieved the largest decreases in TDS and COD, with a pH drop to 3.5 and conductivity of 2443 µmhos/cm	Koosdaryani et al. (2019)
Coagulation-flocculation: Moringa (*M. oleifera*)^*^ Phytoremediation: Indian shot (*Canna indica* L.)^**^	*Seed; (2) **Living plant	Raw leachate landfill	Plants in the vertical sub-surface flow constructed wetland (VSSFCW) system effectively improve landfill leachate quality. The system achieves a 92.88% turbidity reduction, 98.22% Mn removal, and 88.64% COD reduction. The treated effluent has a turbidity of 4.97 mg/L, Mn level of 0.12 mg/L, and COD of 66.36 mg/L, all meeting wastewater quality standards	Rahmadyanti et al. (2021)

*Scientific names are verified through database resources on websites (1) https://powo.science.kew.org/, (2) https://identify.plantnet.org/, and (3) https://www.gbif.org/. *TDS* total dissolved solids, *TSS* total suspended solids, *BOD* biochemical oxygen demand, *COD* chemical oxygen demand, *NTU* nephelometric turbidity units, *FNU* formazin nephelometric unit

**Fig. 2:**
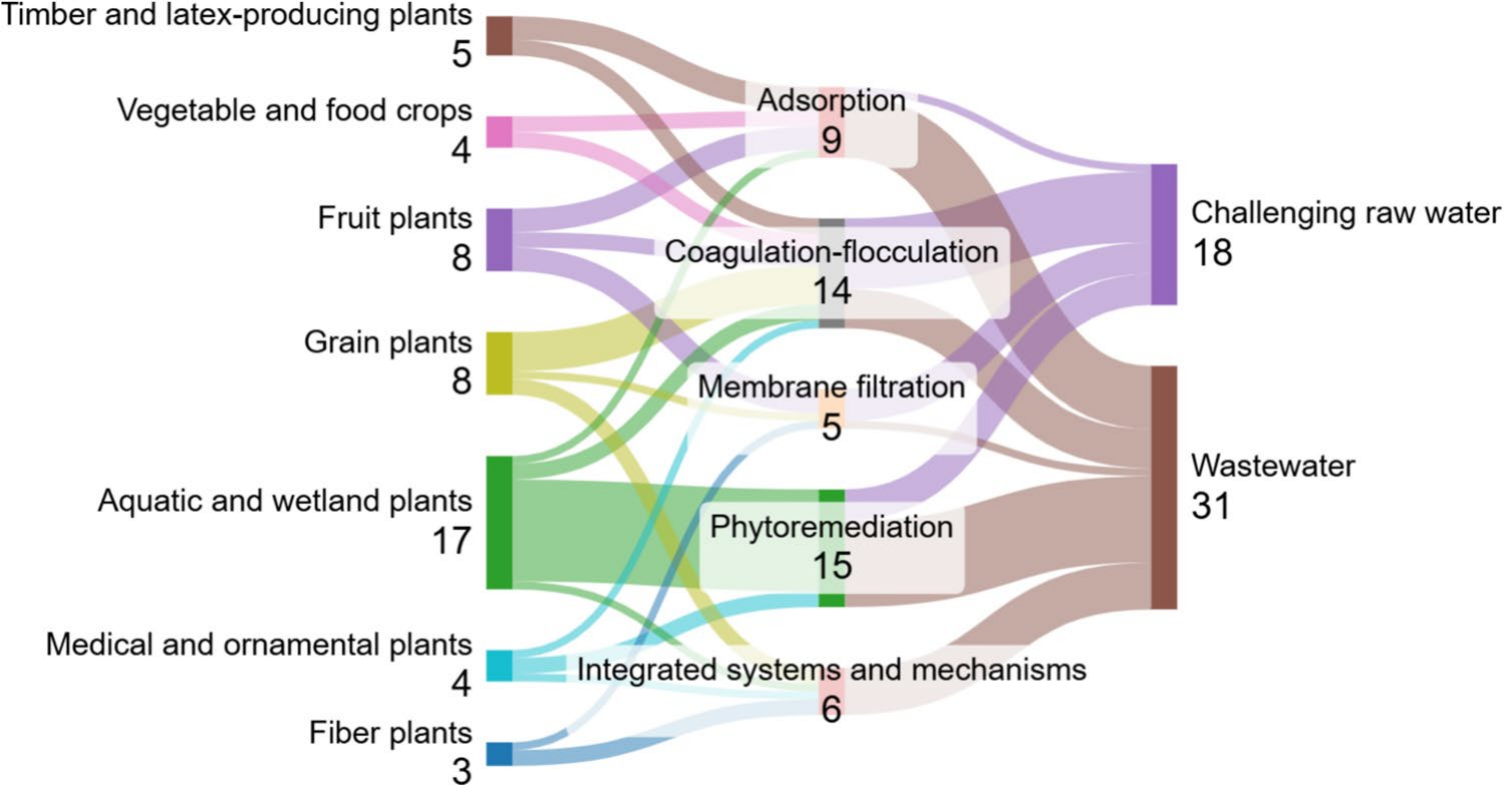
The relationship among the type of plant, the type of treated water, and the mechanisms in the PbWT

The 39 existing research studies focused on raw water treatment (*N* = 15) and wastewater treatment (*N* = 25), with one study having a dual focus on raw water and wastewater in one article (Hendrawati et al. 2016). Additionally, there were four studies that used more than one type of plant in their research (Radityaningrum & Kusuma 2017; Koosdaryani et al. 2019; Henny et al. 2019; Rahmadyanti et al. 2021). In these discussions, two studies considered more than one type of plant for treatment (Radityaningrum and Kusuma 2017; Henny et al. 2019), and one study examined treatment involving several mechanisms (Koosdaryani et al. 2019). The coding and visualization of findings in the literature indicate that the predominant types of plants are aquatic and wetland species. The mechanisms frequently discussed relate to phytoremediation and coagulation-flocculation, while the processing primarily focuses on wastewater, as illustrated in Fig. 2.

## Discussion

The findings presented in Tables 3 and 4 indicate that Indonesia is rich in local plant species that could effectively support plant-based water treatment methods to address challenging raw water and wastewater. Various processes are involved in the treatment of water from different sources. Sustainable water treatment highlights the need to understand the properties of water and wastewater from different activities or industries to choose suitable treatment methods (Kato and Kansha 2024). This discussion section will further explore potential types of plants, the possible mechanisms involved in the treatment process, the types of challenging raw water or wastewater that require treatment, and the challenges and future directions for implementing local plant-based water treatment in Indonesia. It will also address how these methods can support environmental sustainability.

### Types of Indonesia’s local plants with potential in water and wastewater treatment processes

#### Aquatic and wetland plants

They are the dominant groups that are often used in water treatment in Indonesia. The reviewed document includes eleven types of plants: duckweed, watermilfoil, vetiver, heliconia, mangrove, water hyacinth, cattail, azolla, lemna, papyrus, and bulrush (Harbowo and Choesin 2014; Titah et al. 2016; Radityaningrum and Kusuma 2017; Ahmad et al. 2017; Effendi et al. 2017; Koosdaryani et al. 2019; Henny et al. 2019; Prihatinningtyas 2019, 2020; Noor et al. 2020; Cundari et al. 2023; Nooryaneti et al. 2023). These plants can play an important role in adsorbing pollutants and nutrients from the aquatic environment.

#### Fruit plants

Fruit plants such as orange peel, banana, papaya, areca nut, coconut, and oil palm are widely suggested in wastewater treatment. Parts of these plants, such as peel or fiber, have effective adsorptive properties to attract and reduce contaminant content and were used in eight applications (Yudanto et al. 2016; Allwar et al. 2019; Novallyan et al. 2021; Zainol et al. 2022; Pramono et al. 2022; Elma et al. 2022; Widiyanti et al. 2023; Wilanda et al. 2023).

#### Fiber plants

Fiber plants, particularly kapok, sugar cane, and bamboo, are commonly used in various applications (Koosdaryani et al. 2019; Rusdi et al. 2023)*.* They play a significant role in coagulation and filtration processes, serving as absorbents or filters for large particles and organic materials. Specifically, sugar cane and bamboo have been utilized in an integrated system.

#### Grain plants

Grain plants such as moringa, winged bean, soybean, rice, and mung bean are also widely used, as seen in eight applications, two of which are used in integrated systems (Hendrawati et al. 2016; Sisnayati et al. 2019; Koosdaryani et al. 2019; Maulidya and Putra 2020; Putra et al. 2021b; Rahmadyanti et al. 2021; Istiqomah et al. 2023). These grains are often relied upon as natural coagulants that are effective in reducing water turbidity, especially in domestic or industrial waste.

#### Medicinal and ornamental plants

Medicinal and ornamental plants, such as aloe vera, tiger lilies, parrot’s beak heliconia, and Indiana shot provide additional contributions in reducing certain contaminants and maintaining water quality, with a total of four suggested applications utilizing the natural properties of these plants in treating water, one of which is used in integrated systems (Rahmadyanti et al. 2020, 2021; Putra et al. 2021a; Sutanto and Bawole 2021).

#### Timber and latex-producing trees

Timber and latex-producing trees, such as agarwood, rubber trees, kusambi, leucaena, and laban, in five applications (Fenditasari et al. 2019; Kristianto et al. 2019; Neolaka et al. 2020; Sri Hartati et al. 2021; Zulfikar et al. 2020). These plants are often chosen because of the ability of their wood and seeds to adsorb organic and inorganic substances in water.

#### Vegetables and food crops

These plants were applied in four studies. Vegetables and food crops, including Chinese cabbage and sweet potatoes, provide additional benefits with high adsorption capacity, although they are used in more limited quantities (Alni et al. 2019; Kusuma et al. 2021; Rohana and Asmoro 2020). Another type of plant we include in this category is organic starch-producing plants, specifically sago (Fauzia et al. 2019), which serves as a primary food source providing carbohydrates, like sweet potatoes.

### Mechanisms involved in the treatment process

Water treatment aims to meet high hygiene standards for human consumption, while wastewater treatment focuses on eliminating hazardous substances so they can be safely discharged or reused. Wastewater typically contains organic matter, heavy metals, dyes, and pathogens, necessitating coagulation, adsorption, and phytoremediation to bind and decompose these pollutants. In contrast, uncontaminated water that needs filtration usually contains only minor impurities, minerals, or microorganisms, which can be removed through filtration and sterilization. Thus, the production of clean water is held to stricter quality standards for safe consumption, whereas wastewater treatment emphasizes safe disposal. Environmental safety considerations in water and wastewater treatment reflect the goal of developing processes that are more sustainable and beneficial for both the environment and human health (Mohamed Noor and Ngadi 2024).

Water treatment aims to remove contaminants from polluted water, including colloidal particles, pathogens, suspended molecules, and other toxic materials that can harm human health. The treatment comprises two stages: the primary stage employs sedimentation and filtration to mechanically remove solid particles, while the secondary stage employs biological agents (anaerobic or aerobic microorganisms) to break down and remove remaining waste and minute particles. Water treatment methods include chemical, physical, and biological techniques (Koul et al. 2022). Chemical methods encompass coagulation, ion exchange, disinfection, catalytic reduction, oxidation, and softening processes (Guo et al. 2020; Alibeigi-Beni et al. 2021). Physical methods include adsorption, UV processes, settling, and media and membrane filtration (Ali and Gupta 2006; O’Malley et al. 2020). Biological methods involve phytoremediation, bioreactor processes, microbial biodegradation, and wetlands (Ang and Mohammad 2020). A combination of these methods is often used to enhance efficiency (Hamzah et al. 2017; Koosdaryani et al. 2019; Rahmadyanti et al. 2020, 2021; Nimesha et al. 2022). The categorization presented in Tables 3 and 4 indicates that the mechanisms underlying water treatment using Indonesia’s local plants are related to adsorption, coagulation-flocculation, membrane filtration, and phytoremediation.

#### Adsorption

Removing metals, non-metals and small particulates from a solution by adsorption mechanism through any biological component is known as biosorption. Researchers have demonstrated the biosorption potential of various Indonesian plants, including agarwood (Hartati et al. 2021), areca fiber waste (Novallyan et al. 2021), banana fruit bunches (Allwar et al. 2019), Chinese cabbage (Alni et al. 2019), sago bark (Fauzia et al. 2019), rubber seeds (Zulfikar et al. 2020), Kusambi wood (Neolaka et al. 2020), orange peels (Zainol et al. 2022), and water hyacinth (Cundari et al. 2023). Tables 3 and 4 highlight how these plant parts are converted into high-carbon adsorbents, indicative of their adsorption capacity. Cellulose, hemicellulose, and lignin are primary components in grain-based products, with proportions varying by product. For instance, rice-based biomass contains 32.24% cellulose, 21.34% hemicellulose, and 21.44% lignin, while wheat-based biomass has 39% cellulose, 35% hemicellulose, and 14% lignin (Demirbas 2008; Farooq et al. 2010). Cellulose is known to adsorb phenol (Alni et al. 2019) and humic acid (Pramono et al. 2022). Cellulose and hemicellulose in agricultural and plant biomass enhance their biosorption potential. Other agricultural byproducts like tea, coffee, shells, nuts, and various fruit seeds also contain cellulose, hemicellulose, and lignin (Mathew et al. 2016).

Interactions between water pollutants and biosorbents occur via surface sorption and interstitial sorption. In surface sorption, sorbate molecules move from the solution to the biosorbent’s surface, attaching to active sites through dipole interactions, hydrogen bonds, or Van der Waals forces (Sulyman et al. 2017). In interstitial sorption, pollutants diffuse into the biosorbent’s pores and attach to the inner surface (Joseph et al. 2019). Electrostatic interactions also play a significant role in adsorbing water contaminants. The abundance of functional groups on the surfaces of biosorbents renders them highly effective in capturing pollutants from water systems (Elgarahy et al. 2021).

#### Coagulation-flocculation

Plant-based coagulants are more readily available than coagulants derived from animals or microorganisms. Several plant-based products, including aloe vera (Putra et al. 2021a), papaya (Widiyanti et al. 2023; Wilanda et al. 2023), sweet potato (Rohana and Asmoro 2020; Kusuma et al. 2021), duckweed (Prihatinningtyas 2019, 2020), laban wood (Fenditasari et al. 2019), mung bean (Putra et al. 2021b), soybean (Maulidya and Putra 2020), winged bean (Istiqomah et al. 2023), moringa (Hendrawati et al. 2016), and leucaena (Kristianto et al. 2019) have been employed in the treatment of polluted water. Macromolecules originating from these plants, such as proteins, polysaccharides, and certain functional groups, facilitate adsorption, polymer linking, and charge neutralisation, rendering them effective in water treatment with moderate turbidity levels of 50–500 NTU. The efficacy of natural coagulants can be enhanced through the optimization of the extraction and purification process, thereby improving waste disposal efficiency (Koul et al. 2022).

Coagulation gathers unstable particles into larger groups for separation through sedimentation or filtration. The coagulation-flocculation mechanism involves charge neutralization, polymer bridging, sweep-flocculation, and double-layer compression. Charge neutralization occurs when oppositely charged coagulants adsorb onto colloidal particles, while polymer bridging connects particles with long-chain polymers. Sweep-flocculation takes place when metal coagulants exceed solubility limits, forming amorphous hydroxides, and double-layer compression occurs in high-electrolyte solutions, diminishing repulsive forces. Among these, polymer bridging and charge neutralization are predominant in plant-based coagulants (Yin 2010; Koul et al. 2022).

#### Membrane filtration

Banana peel, containing about 24.8% pectin, enhances the hydrostability and mechanical strength of mesoporous silica membranes in wetland water treatment through ultrafiltration. Adding carbon from pectin to the silica matrix improves hydro stability and water desalination performance (Elma et al. 2022). Rice husk is also used in fabricating ceramic membranes (Sisnayati et al. 2019). Supplementary membrane materials must combust completely, avoiding tar or ash, and create smaller pores than the primary material (clay). Other research has demonstrated the phase inversion method effectively fabricates PVDF (polyvinylidene fluoride) and PVDF/cellulose membranes for treating humic acid water, with cellulose from oil palm fruit bunches available as microcrystalline (MCC) and nanocrystalline cellulose (NCC) (Pramono et al. 2022). Kapok, composed of 64% cellulose, 13% lignin, and 23% pentosan, also shows promise for oil-contaminated water treatment but requires delignification to remove lignin that interferes with metal ion binding (Rusdi et al. 2023). Additionally, coconut fiber, a material from cellulose plant parts, is used in microfiltration with active zeolite filters for treating river water (Yudanto et al. 2016).

This membrane technology employs permeable or semi-permeable barriers to separate contaminants based on size and charge. This process relies on driving forces like pressure and concentration gradients (Waqas et al. 2021; Mustalifah et al. 2021; Isnasyauqiah et al. 2022; Nawi et al. 2022). The separation of contaminants depends on their size and charge. To facilitate movement across the membrane, driving forces such as pressure differences, concentration gradients, and potential fields are necessary. Pressure-driven membrane systems are classified according to their operating pressure. Low-pressure membranes (microfiltration and ultrafiltration) operate at 10–30 psi, while high-pressure membranes (nanofiltration and reverse osmosis) require 75–250 psi (Othman et al. 2021).

#### Phytoremediation

Plants for rhizofiltration should have dense root systems, high biomass, and heavy metal tolerance. Both terrestrial and aquatic plants are useful. Aquatic species like water hyacinth (Noor et al. 2020; Nooryaneti et al. 2023), azolla, duckweed, and cattail (Radityaningrum and Kusuma 2017) are favoured for their heavy metal accumulation, tolerance, fast growth, and biomass. Ornamental plants such as papyrus (Harbowo and Choesin 2014), heliconia (Sutanto and Bawole 2021), and parrot’s beak heliconia (Rahmadyanti et al. 2020) can also treat wastewater and improve water quality. Grasses in swamps, like bulrush (Titah et al. 2016) and vetiver (Effendi et al. 2017), reduce total suspended solids, chemical oxygen demand, and biological oxygen demand in grey water and oil spills. The mangrove ecosystem includes vegetation like *R. mucronate* (Ahmad et al. 2017), effective in treating brackish water and preventing eutrophication and pollution-related disease outbreaks.

**Fig. 3:**
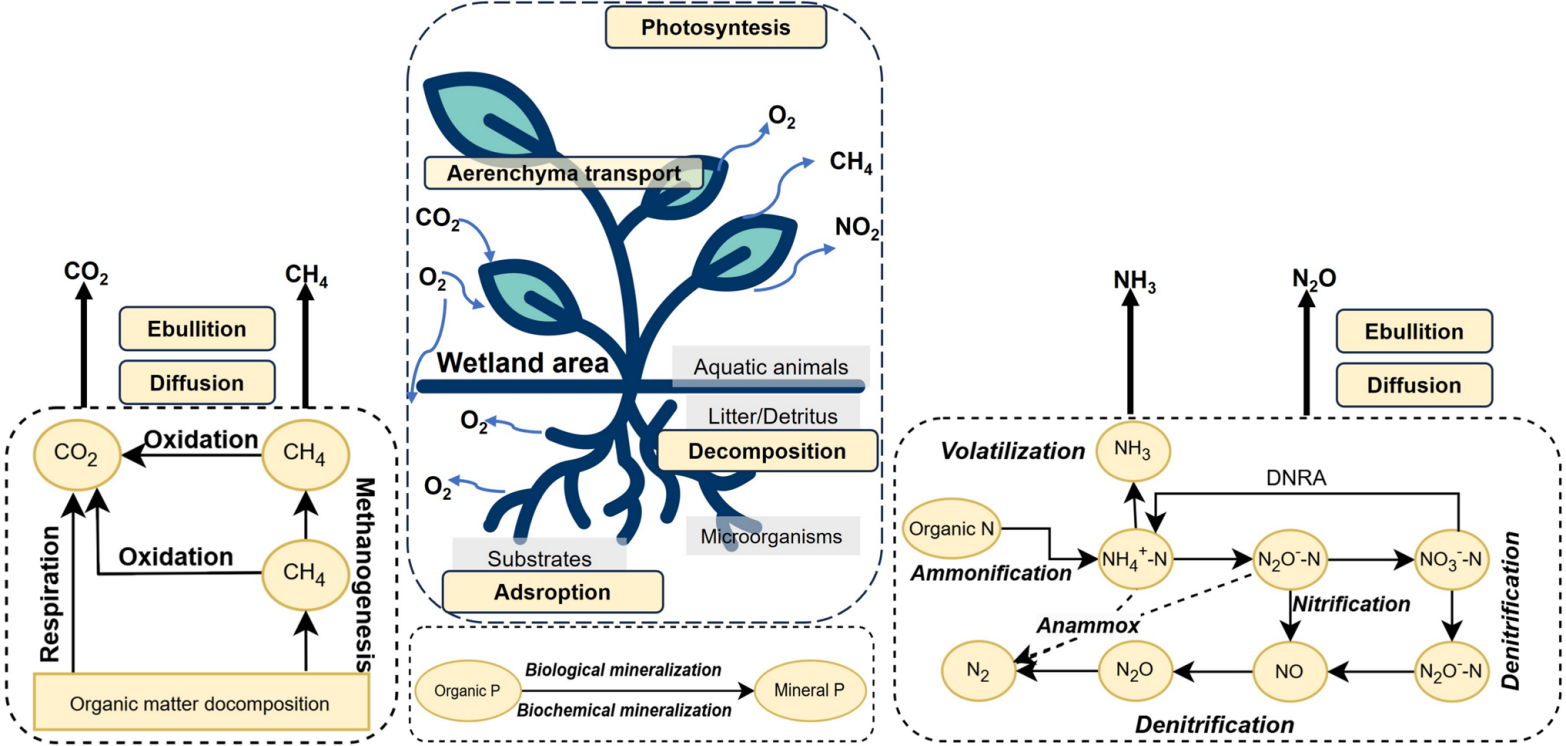
The mechanisms of the phytoremediation process in a constructed wetland (modified from Wu et al. 2023)

Phytoremediation is a plant-based approach that employs plants to extract and remove pollutants or reduce their bioavailability (Raskin et al. 1997; Oladoye et al. 2022). The physical and chemical properties of wetlands create conditions that favour phytoremediation of water pollution and strengthen redox reactions between plants and microorganisms in the rhizosphere (Macek et al. 2000). In order to reduce eutrophication in aquatic ecosystems, plants are used to remove nitrogen and phosphorus from water (Liu et al. 2016; Wei et al. 2021). Figure 3 illustrates the removal of pollutants in wastewater through synergistic interactions between vegetation, microorganisms, aquatic animals, and substrates in constructed wetlands (CWs) (Lee et al. 2009; Wu et al. 2023). The organic matter in CWs is primarily decomposed by bacteria attached to plant roots and filter media. The transformation and removal of nitrogen in CWs encompasses microbial cycling, ammonia evaporation, absorption, desorption, burial, and leaching (Vymazal 2007, 2011). Phosphorus transformation and removal involves adsorption, desorption, precipitation, dissolution, absorption by plants and microbes, leaching, mineralization, sedimentation, and burial (Vymazal 2007). Furthermore, water purification in CWs can result in the production of significant quantities of greenhouse gases through diffusion or the formation of gas bubbles in the water, or active transport by plants (Malyan et al. 2016; Maucieri et al. 2017).

### Types of treated water

#### Challenging raw water

In water treatment, local plants can be utilized to process challenging raw water, making it suitable for consumption or community use. For instance, *peat water* is treated with soybean and mung bean, which act as natural coagulants to precipitate dyes and organic impurities, resulting in clearer and safer water for domestic or agricultural needs (Maulidya and Putra 2020). *Peat water and wetland saline water*, which contains natural organic matter and salt, can be treated using banana peels as an adsorbent. The banana peels bind organic compounds and salts, effectively reducing the levels of these contaminants in both peat water and saline wetland water (Elma et al. 2022). For *river and well water*, areca palm fiber and kapok fiber can serve as adsorbents to remove heavy metals, such as lead (Pb), and *E. coli* bacteria, ensuring that the water is safe for consumption and household use (Novallyan et al. 2021).

#### Wastewater

In the case of *domestic wastewater* from infiltration wells and households, parrot’s beak heliconia, and water hyacinth can be employed as phytoremediators. These plants absorb nutrients and organic matter, significantly reducing turbidity, BOD, phosphate levels, and coliform bacteria. In one study, parrot’s beak heliconia reduced BOD by 71.64%, phosphate by 50.92%, and coliform bacteria by 99.67% from domestic wastewater (Sutanto and Bawole 2021). In *aquaculture effluent* from shrimp pond water, which is prone to eutrophication, mangrove (*R. mucronata*) can be utilized to absorb excess nutrients and organic compounds, helping control pollution and inhibit the growth of harmful bacteria (Ahmad et al. 2017). For *tofu-tempeh and tapioca factory wastewater*, which is rich in organic matter and requires turbidity reduction, winged bean seeds can be used to precipitate particles through natural coagulation. This process produces clearer water and increases dissolved oxygen (DO) levels, making it safer for the environment (Istiqomah et al. 2023).

*Wastewater from the textile industry*, known for high levels of BOD, COD, and total suspended solids (TSS), is processed using moringa seeds through a coagulation-flocculation mechanism, along with agarwood as an adsorbent. The coagulation process of moringa seeds helps to precipitate suspended particles, while agarwood absorbs organic matter and reduces colour in textile wastewater, making it safer for discharge (Hartati et al. 2021). For *dye waste from the batik industry*, which contains oils and dyes, tiger lily can act as a phytoremediator to reduce COD, TSS, oil, and fat, while bamboo charcoal columns effectively adsorb dyes and stabilize the wastewater. This combination reduced COD by 72–86% and TSS by 95–98% (Rahmadyanti et al. 2020). For *wastewater containing heavy metals*, kusambi wood, and sago bark are effective in adsorbing heavy metals like chromium (Cr(VI)). These plants have a high absorption capacity, ensuring that the water is safer for disposal (Neolaka et al. 2020). *Wastewater from oil and gas factories* is treated with vetiver grass, which functions as a phytoremediator in decomposing crude oil and organic compounds through active absorption. Additionally, kapok fiber can be utilized as a membrane filtration medium to filter TDS, phenols, and heavy metals. The treatment results showed a reduction in oil, COD, and BOD by 84–90% (Effendi et al. 2017).

### Challenges and future directions for plant-based water treatment

Tables 3 and 4 outline various plants with the potential to be used in treating water and wastewater, particularly in laboratory studies. Plants like Chinese cabbage, rubber seeds, oranges, and bananas, along with various plant parts, effectively removed simple pollutants such as phenols and heavy metals from artificial wastewater. However, these results may not fully represent the actual complexity of wastewater treatment. Pilot projects can assess their effectiveness in small-scale facilities using real wastewater. Successful trials could lead to large-scale implementation, requiring methods for regenerating materials and managing pollutant variations. Plants such as agarwood and winged bean seeds have proven effective in reducing organic pollutants and dyes in specific contexts like textiles and tofu-tempeh production. Further small-scale pilot projects are necessary to explore their potential, and larger systems will be needed for comprehensive management in industrial settings.

Plants such as papaya, sweet potato, duckweed, and water hyacinth are effective in treating domestic wastewater and river water that contain low to moderate concentrations of organic pollutants. These plants have demonstrated positive results in improving water quality by reducing TSS, pH, and COD. However, domestic wastewater treatment in Indonesia faces challenges due to high volumes of untreated greywater and insufficient treatment infrastructure at the household level (Widyarani et al. 2022). In pilot projects, constructed wetlands or simple biofiltration systems can be tested in residential areas or near rivers to evaluate the potential adaptation of these plants to real-world conditions. Implementing plant-based water and wastewater treatment on a large scale, particularly through constructed wetland systems like vertical sub-surface flow constructed wetlands, involves several considerations. These include the requirement for large areas, routine maintenance of biomass, effectiveness at the community level, and management of residuals. In another case, for treating oil and mining wastewater, plants such as vetiver and bulrush have proven highly effective in absorbing heavy metals and organic compounds. These plants are suitable for phytoremediation in complex environments, including mining sites or oil-contaminated land. Pilot projects can be initiated by planting these species in polluted areas or wastewater retention ponds to evaluate their effectiveness under field conditions. While large-scale application at mining sites or oil industries may be feasible using constructed wetland systems, it necessitates close monitoring, as oil-polluted environments can hinder plant growth and absorption capabilities. Well-designed and properly managed constructed wetlands have great potential to enhance biodiversity and strengthen their ability to provide sustainable and environmentally friendly wastewater treatment solutions (Hsu et al. 2011). Wetlands’ effectiveness in treating industrial wastewater relies on the complex interactions between plants and microbial communities, which play a crucial role in biodiversity. Choosing the right plants and using strategies like bacterial inoculation and co-planting certain species can enhance treatment efficiency, resulting in environmentally friendly and sustainable solutions (Mao et al. 2023).

Plant materials such as coconut fiber, rice husk, and kapok fiber, which are used for filtration membranes, have significant potential for water filtration. Research has demonstrated that these materials reduce TDS, BOD, COD, and dyes at the laboratory scale. For pilot projects, these plant-based membranes can be evaluated in facilities with low waste volumes, such as workshops or small laboratories, to assess the durability of the materials. On a larger scale, producing plant-based filtration membranes requires technical adjustments concerning the material’s service life and regeneration capabilities. However, these membranes can offer a practical solution in resource-limited areas, particularly where modern or expensive filtration technologies are unavailable.

Keeping plant biodiversity can play a vital role in sustainable water and wastewater treatment. Jain et al. (2023) indicated that biomaterials from different sources have high adsorption capacity and regeneration potential, which support water treatment with minimal ecological impact. Koul et al. (2022) also highlighted that environmentally friendly bio-based natural coagulants can strengthen sustainability principles in water treatment, support the circular economy, and improve public health. In addition, Tripti et al. (2023) revealed that using carbon dot-based photocatalysts derived from biomass shows the potential of bio-materials in offering more economical and non-toxic water treatment solutions. While early research shows promise, more field trials are necessary to confirm the effectiveness of using local plant material for wastewater treatment on a larger scale. With the support of local regulations and infrastructure, implementing plant-based solutions can offer a cost-effective and environmentally friendly option for managing water and industrial wastewater, particularly in communities seeking sustainable alternatives. Each plant species has unique capabilities to remove or break down specific substances found in wastewater, highlighting the value of plants in pursuing environmental sustainability and clean water solutions.

## Conclusion

Using local plants in water and wastewater treatment has the potential to provide effective and sustainable solutions for removing pollutants. Different types of plants, such as aquatic and wetland plants, fruit and fiber plants, grains, medicinal and ornamental plants, and timber trees, offer mechanisms like adsorption, coagulation, and phytoremediation. These mechanisms can help reduce contaminants such as heavy metals, organic compounds, pathogens, and dyes.

Plants are utilized across different water types, including peat water, agricultural runoff, and both industrial and domestic wastewater. This illustrates their adaptability and effectiveness in improving water quality. However, although laboratory results indicate significant potential for using local plants in water and wastewater treatment, the large-scale implementation of this method faces several challenges. For instance, these include the need for improved infrastructure for domestic water treatment at the household level, along with the complexities of industrial waste management.

Plant-based water and wastewater treatment has inherent limitations, including efficiency variations, dependency on environmental conditions, and the potential generation of secondary pollutants. To move forward, it is essential to develop larger, community-based pilot projects that integrate plant-based water treatment within specific socio-ecological contexts. Further research is necessary to evaluate the effectiveness of various local plants in addressing complex and diverse types of water pollution on a larger scale. Additionally, challenges related to plant material regeneration, system maintenance, and the management of treatment residues must be addressed to ensure that plant-based solutions can be implemented sustainably. With appropriate regulatory and infrastructure support, plant-based water treatment solutions can serve as a cost-effective and environmentally friendly alternative, contributing to the sustainability of water resources in Indonesia.

## Limitation

This study has three main limitations. First, it focuses solely on English-language publications, potentially underrepresenting contributions from non-English-speaking countries and Indonesian water treatment research published in other languages. Second, it only includes journal articles, book chapters, and conference proceedings, excluding other publication types. Third, no additional data searches were conducted beyond those in the three databases (Scopus, WoS, and Google Scholar).

## Data Availability

This study is a literature review and does not involve collecting primary data. The information supporting this review was gathered from metadata and publications indexed in databases such as Scopus, Web of Science, and Google Scholar. Access to these databases is subject to their respective subscription and access policies.
